# The Synthesis, Structural Characterization, and DFT Calculation of a New Binuclear Gd(III) Complex with 4-Aacetylphenoxyacetic Acid and 1,10-Phenanthroline Ligands and Its Roles in Catalytic Activity

**DOI:** 10.3390/molecules29133039

**Published:** 2024-06-26

**Authors:** Ying Liu, Xiao Tang, Xi-Hai Yan, Li-Hua Wang, Xi-Shi Tai, Mohammad Azam, Dong-Qiu Zhao

**Affiliations:** 1 College of Chemistry and Chemical Engineering, Weifang University, Weifang 261061, China; 2 College of Science, Institute of Materials Physics and Chemistry, Nanjing Forestry University, Nanjing 210037, China; 3 College of Biology and Oceanography, Weifang University, Weifang 261061, China; 4 Department of Chemistry, College of Science, King Saud University, Riyadh 11451, Saudi Arabia; 5 School of Physics and Electric Engineering, Anyang Normal University, Anyang 455000, China

**Keywords:** Gd(III) complex, synthesis, crystal structure, Hirschfeld surface analysis, DFT, photocatalytic CO_2_ reduction activity, oxidation of benzyl alcohol

## Abstract

A new binuclear Gd(III) complex, [Gd_2_(L)_6_(Phen)_2_]·4H_2_O, was synthesized via the reaction of gadolinium(III) nitrate hexahydrate, 4-acetylphenoxyacetic acid (HL), NaOH, and 1,10-phenanthroline (Phen) in a solution of water–ethanol (*v*:*v* = 1:1). The Gd(III) complex was characterized using IR, UV–vis, TG-DSC, fluorescence, and single-crystal X-ray diffraction analyses. The results showed that the Gd(III) complex crystallizes in the triclinic system, space group *P*-1, and each Gd(III) ion was coordinated with two nitrogen atoms (N1, N2, or N1a, and N2a) from two Phen ligands and seven oxygen atoms (O1, O2, O7a, O9, O8, O8a, O10a, or O1a, O2a, O7, O8, O8a, O9a, and O10) from six L ligands, respectively, forming a nine-coordinated coordination mode. The Gd(III) complex molecules formed a one-dimensional chained and three-dimensional network structure via benzenering π-π stacking. The Hirschfeld surface analysis and the calculations of the electron density distributions of the frontier molecular orbitals of the Gd(III) complex were performed. The catalytic activities of the photocatalytic CO_2_ reduction and benzyl alcohol oxidation using the Gd(III) complex as a catalyst were performed. The results of the photocatalytic CO_2_ reduction showed that the yield and the selectivity of CO reached 41.5 μmol/g and more than 99% after four hours, respectively. The results of the benzyl alcohol oxidation showed that the yield of benzaldehyde was 45.7% at 120 °C with THF as the solvent under 0.5 MPa O_2_ within 2 h.

## 1. Introduction

Photocatalytic CO_2_ reduction technology with metal semiconductors, boron nitride derivatives, metal sulfide and carbon-based materials as catalysts has attracted wide attention from material scientists and chemists because they can convert CO_2_ into high value-added products, such as methane, CO, methanol, ethanol, and formic acid [1,2,3,4,5,6,7,8,9,10,11,12]. For example, the Rh/Al_2_O_3_ catalyst can achieve a methane selectivity above 98% [7]. The nickel–boron nitride catalyst has a high CO_2_ conversion rate (87.68%), high CO_2_ catalytic rate (2.03 mol gNi^−1^h^−1^), high stability, and high CH_4_ selectivity (99.83%) [8]. Magnetic-field-regulated TiO_2_ can enhance the coupling of the CO* intermediates, increasing the yield rate of CO_2_ to C_2_H_5_OH 22-fold higher than pristine TiO_2_ [9]. The CO production rate is 28.83 μmol·g^−1^·h^−1^ with the NALDH/CN/GA-20 hybrid system as a catalyst, which is 24 and 16 times that of pure NALDH and bare CN, respectively [11]. However, the above catalyst materials have disadvantages, such as expensive costs and difficulty in determining the composition. Benzaldehyde is an important organic raw chemical material that is widely used in the chemical industry, dairy products, cellulose and synthetic fiber materials, medicine, and other fields. However, benzaldehydes are prepared via benzyl alcohol oxidation with toxic metal oxides, peroxides, halides, and so on [13,14,15]. Therefore, the research and development of cheap and environmentally friendly catalysts is very urgent. Over the past ten years, metal complexes have garnered considerable attention as catalysts in photocatalytic CO_2_ reduction and benzyl alcohol oxidation reactions due to their tunable properties, simple synthesis, and catalytic activity. Some metal complexes, such as the Co(II) complex [16,17], Mn(II) complex [18], Cu(I) complexes [19], Fe(II) complex [20], Ru(II) complex [21], and Ln(III) complexes [22,23], have shown excellent catalytic activity and selectivity in photocatalytic CO_2_ reduction. For example, the Co(II) complex catalyst shows high photocatalytic CO2 reduction activity with a catalytic rate of 3012.5 μmol·g^−1^·h^−1^ [17]. The Fe (II) complex catalyst exhibits high CO_2_ selectivity (95%) [20]. Some metal complexes, such as the Mn(II) complex [24], Co(II) complex [25], Ru(III) complex [26], Cu(II) complex [27], and Pd(II) complex [28], also exhibit excellent catalytic activity and selectivity in benzyl alcohol oxidation. However, metal complexes have been rarely studied as catalysts for photocatalytic CO_2_ reduction and benzyl alcohol oxidation reactions. In recent years, our research group has been working on photocatalytic CO_2_ reduction and benzyl alcohol oxidation with metal complexes as catalysts [22,23,29,30,31]. For example, we synthesized a new nine-coordinated Yb(III) complex with 4-acetylphenoxyacetic acid and 1,10-phenanthroline ligands, and it showed good photocatalytic activity with the yield and selectivity of CO being 146 mol/g and 97.9% after three hours, respectively [22]. The effect of the solvent and reaction time on the benzyl alcohol oxidation activity of the Ba(II) complex catalyst was studied [31].

**Figure 1:**
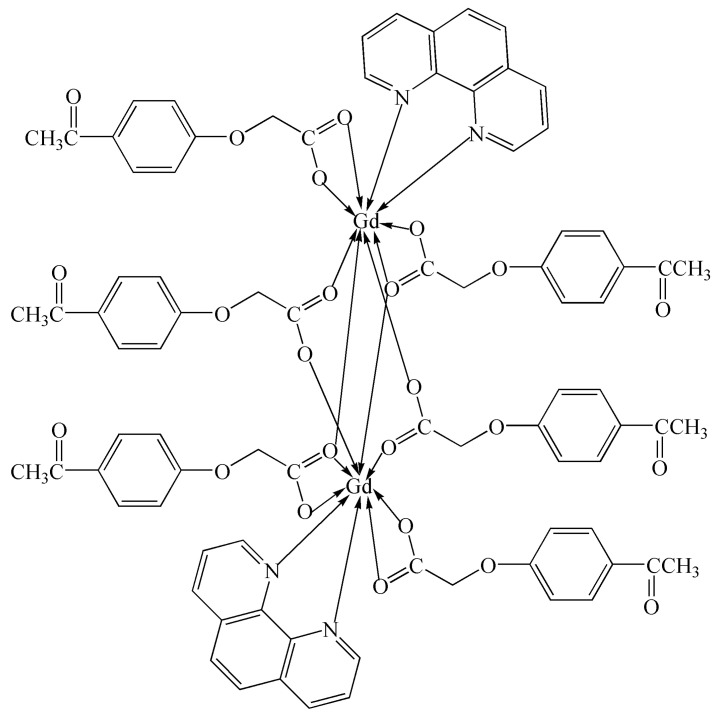
The scheme of the Gd(III) complex.

To further investigate the effects of different central metal ions on the activity and selectivity of the photocatalytic CO_2_ reduction of the complex and further search for the best complex catalyst for catalyzing benzyl alcohol oxidation, we conducted this study. A new binuclear Gd(III) complex was synthesized using gadolinium(III) nitrate hexahydrate, 4-acetylphenoxyacetic acid, NaOH, and 1,10-phenanthroline in a water–ethanol solution. The Gd(III) complex was characterized using IR, UV–vis, TG-DSC, fluorescence, and single-crystal X-ray diffraction analyses, and each Gd(III) ion was coordinated with two nitrogen atoms from two Phen ligands and seven oxygen atoms from six L ligands, respectively, forming a nine-coordinated coordination mode. The Hirschfeld surface analysis and the calculations of the electron density distributions of the frontier molecular orbitals of the Gd(III) complex were performed. The catalytic activities of photocatalytic CO_2_ reduction and benzyl alcohol oxidation using the Gd(III) complex as a catalyst were performed. The results of photocatalytic CO_2_ reduction showed that the yield and the selectivity of CO reached 41.5 μmol/g and more than 99% after four hours, respectively. The results of benzyl alcohol oxidation showed that the yield of benzaldehyde was 45.7% at 120 °C with THF as the solvent under 0.5 MPa O_2_ within 2 h. The scheme of the Gd(III) complex is shown in Figure 1.

## 2. Results and Discussion

### 2.1. Infrared Spectrum

**Figure 2:**
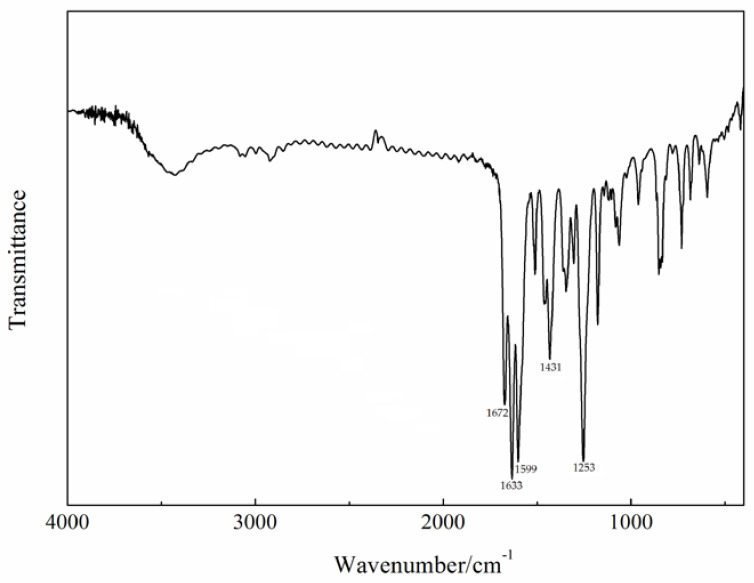
The infrared spectrum of the Gd(III) complex.

The infrared spectrum of the Gd(III) complex is shown in Figure 2. The characteristic bands of free 4-acetylphenoxyacetic acid (HL) are at ca. 1758 (ν(C=O)), 1648 (ν_as_(COO^−^)), 1598 (ν_s_(COO^−^)), and 1286 (ν(C=O)) cm^−1^, respectively [22]. In the Gd(III) complex, they appear at 1672, 1633, 1431, and 1253 cm^−1^. This shows that the carboxylate O atoms of the L ligand are involved in coordination with the Gd(III) ion. The band at 1599 cm^−1^ in the Gd(III) complex is assigned to ν(C=N) of the Phen ligand, indicating that the N atoms of the Phen ligand are also coordinated with the Gd(III) ion.

### 2.2. UV–Vis Spectra

**Figure 3:**
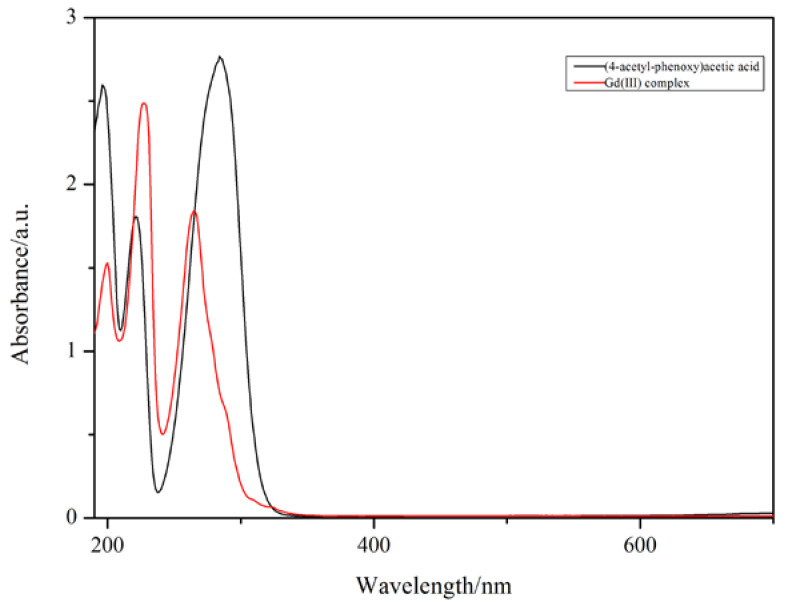
The UV–vis spectra of the Gd(III) complex (red, 1.6 × 10^−5^ mol·L^−1^) and 4-acetylphenoxyacetic acid (HL) (black, 1.8 × 10^−5^ mol·L^−1^). The path length is 1 cm in quartz cuvettes.

The UV–vis spectra of the Gd(III) complex and 4-acetylphenoxyacetic acid (HL) are illustrated in Figure 3. 4-acetylphenoxyacetic acid (HL) shows three absorption bands at 284, 221, and 196 nm, and the Gd(III) complex also shows three absorption bands at 265, 227, and 200 nm, which can be assigned to the π-π* transitions of the 4-acetylphenoxyacetate and Phen ligands. The absorption bands of the Phen ligand are at 323, 264, and 237 nm [32]; however, in the Gd(III) complex, the absorption band at 323 nm does not appear, indicating that the Phen ligand takes part in coordination with the Gd(III) ion.

### 2.3. Thermogravimetric Analysis

**Figure 4:**
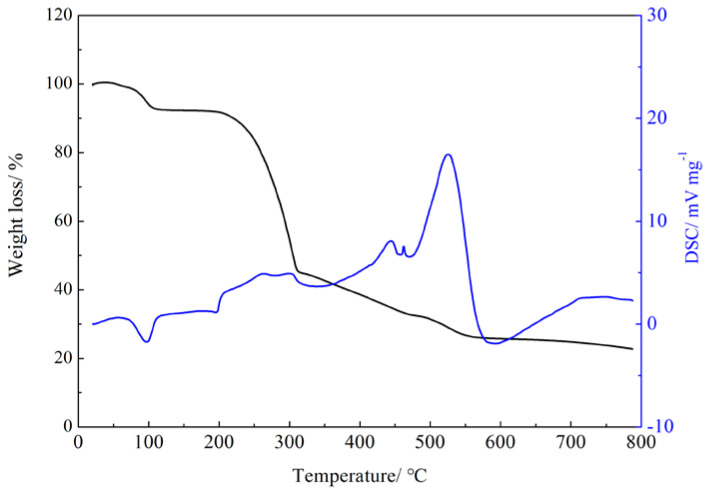
Thermal stability curve of the Gd(III) complex.

To investigate the thermal stability of the Gd(III) complex, the thermogravimetric analysis of the Gd(III) complex was performed at a heating rate of 5 °C/min in an air atmosphere using Al_2_O_3_ as a reference. The thermal stability curve of the Gd(III) complex is shown in Figure 4. The Gd(III) complex shows an endothermic peak at 98 °C, and the weight loss is 5.77% (calculated: 3.78%), which corresponds to the loss of four lattice water molecules. The Gd(III) complex shows three exothermic peaks at 195–325 °C, 423 °C, and 526 °C, indicating that the Gd(III) complex decomposes step by step and continues to lose weight by 80.2%, which corresponds to the decomposition of the 4-acetylphenoxyacetate ligands and Phen ligands.

### 2.4. Structural Description of the Gd(III) Complex

**Figure 5:**
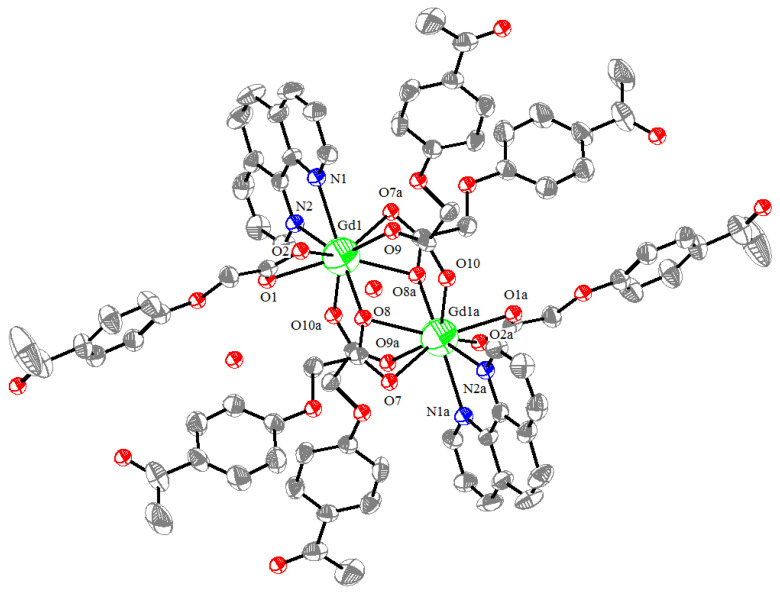
The molecular structure of the Gd(III) complex, symmetry code: a:-*x*, 1-*y*, 1-*z*.

**Figure 6:**
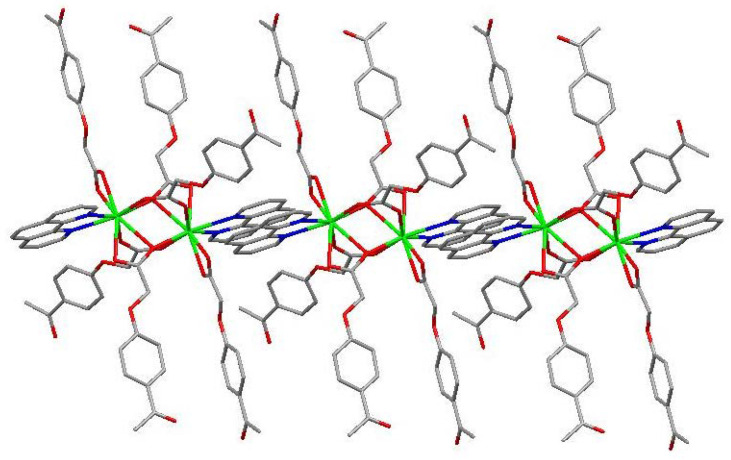
The one-dimensional chained structure of the Phen rings via π-π stacking interactions.

**Figure 7:**
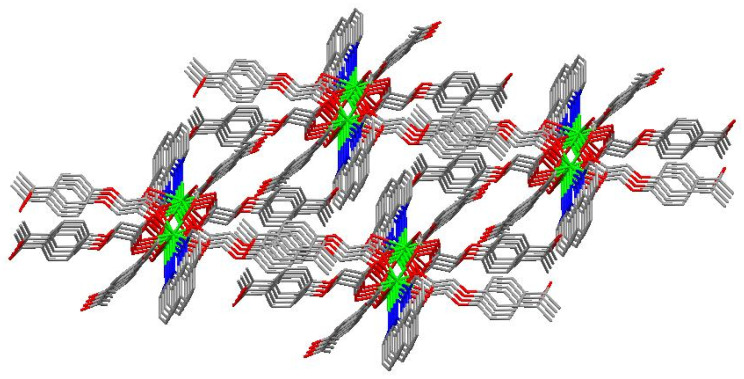
The three-dimensional network structure of the interactions via π-π stacking.

The molecular structure of the Gd(III) complex is shown in Figure 5. Important bond lengths (Å) and angles (°) for the Gd(III) complex are listed in Table 1. The one-dimensional chained structure is shown in Figure 6. The three-dimensional network structure is shown in Figure 7. The single-crystal X-ray diffraction analysis indicates that the structure of the Gd(III) complex is the same as the one we reported for the Yb(III) complex in the literature [22]. Furthermore, the title Gd(III) complex is binuclear according to the bridged oxygen atoms from the carboxylate groups with the Gd(III)-Gd(III) distance of 3.940 Å and crystallizes in the triclinic space group *P*-1. It can be seen from Figure 5 that the Gd(III) complex is made up of two Gd(III) ions, six 4-acetylphenoxyacetate ligands, two Phen ligands, and four lattice water molecules. Each Gd(III) ion is coordinated with three oxygen atoms (O7a, O8a, O8, or O7, O8a, and O8) from two bridged tridentate L ligands, two oxygen atoms (O9, O10a, or O9a, and O10) from two bridged bidentate L ligands, two oxygen atoms (O1, O2, or O1a, and O2a) from one bidentate L ligand, and two nitrogen atoms (N1, N2, or N1a, and N2a) from one 1,10-phenantroline co-ligand. This forms a nine-coordinated environment. The Gd1-O and Gd1-N bond lengths are in the range of 2.350(3)–2.548(3) Å and 2.545(4)–2.599(4) Å, respectively, which is similar to the corresponding bond lengths of those reported in the literature [23]. The O-Gd-O, O-Gd-N, and N-Gd-N bond angles lie in the range of 52.44(11)–148.81(11)°, 72.45(11)–148.26(12)°, and 63.96(12)°, respectively. The angles between the two Gd(III) ions with the two bridged oxygen atoms (Gd1-O8a-Gd1a or Gd1-O8-Gd1a) are 106.57°. The Gd(III) complex molecules form a one-dimensional chained structure (Figure 6) and a three-dimensional network structure via π-π stacking interactions (Figure 7 and Table 2). The π-π stacking interaction data of the Gd(III) complex is listed in Table 2.

### 2.5. DFT Computation

**Figure 8:**
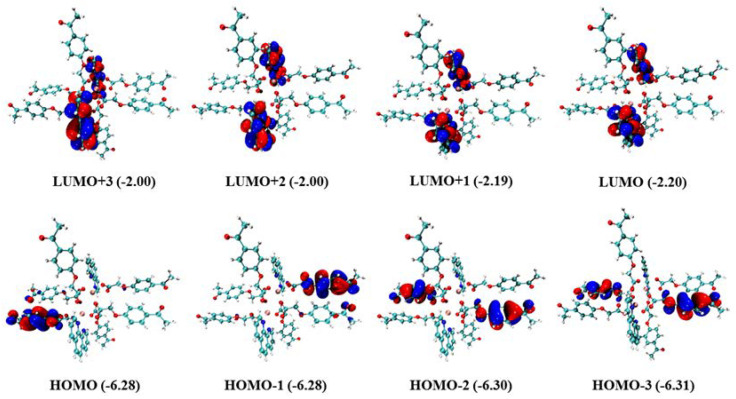
Electron density distributions and energy levels (eVs) of the frontier molecular orbitals for the Gd(III) complex (isovalue = 0.05 e·bohr^−3^).

The electron density distributions and energy levels (eVs) of the frontier molecular orbitals for the Gd(III) complex are provided in Figure 8. As shown in Figure 8, the electron densities of HOMO-3, HOMO-2, HOMO-1, and HOMO are located on different 4-acetylphenoxyacetate ligands, whereas LUMO, LUMO + 1, LUMO + 2, and LUMO + 3 are located on the Phen ligand. Moreover, there are nearly the same energy levels for LUMO, LUMO + 1, and other corresponding frontier molecular orbitals, which indicates that they are degenerate frontier molecular orbitals. The absorption of the two ligands, HL and Phen, was calculated with the time-dependent DFT (TDDFT) at the theoretical level of B3LYP/6-31G* [33], in which the added hydrogen atom on HL is optimized with the other fixed atoms. The calculated absorption spectrum is shown in Figure S1, and it correlates well with the experimental results despite somewhat hypsochromic shifts. It confirms that the absorption spectrum of the Gd(III) complex resembles those of the two ligands.

### 2.6. Hirschfeld Surface Analysis of the Gd(III) Complex

**Figure 9:**
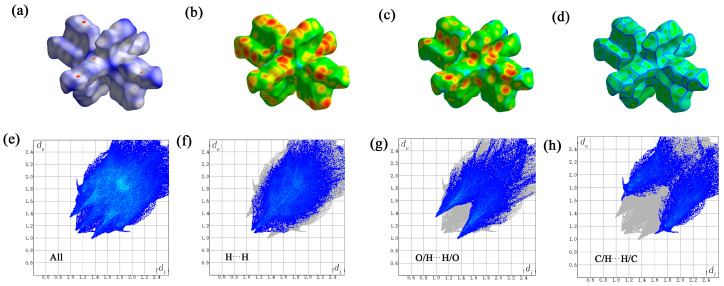
The Hirschfeld surface of the Gd(III) complex. the dnorm (**a**), di (**b**), de (**c**), and crystal’s roughness depicted in panels (**d**), all interactions (**e**), hydrogen–hydrogen (**f**), oxygen–hydrogen/hydrogen-oxygen (**g**), and carbon–hydrogen/hydrogen–carbon interactions (**h**).

Crystal Explorer software (version 21.5) was utilized to examine the Hirschfeld surface of the Gd(III) complex. Figure 9 illustrates the Hirschfeld surfaces, with the dnorm, di, de, and crystal’s roughness depicted in panels a to d. Additionally, the two-dimensional fingerprint plots are displayed, which provide a comprehensive view as well as detail the top three types of interactions: all interactions, hydrogen–hydrogen, oxygen–hydrogen/hydrogen-oxygen, and carbon–hydrogen/hydrogen–carbon interactions, as shown in panels e to h. According to the computational analysis, hydrogen–hydrogen contacts were identified as the predominant factor, accounting for 39.4% of the Hirschfeld surface area. Oxygen–hydrogen/hydrogen made substantial contributions to the manuscript and cannot be changed–made substantial contributions to the manuscript and cannot be changed oxygen interactions and carbon–hydrogen/hydrogen made substantial contributions to the manuscript and cannot be changed–carbon interactions followed, with contributions of 29.9% and 24.9%, respectively. Notably, π-π stacking interactions, as indicated by the carbon–carbon contacts, minimally contributed to the crystal structure, with a Hirschfeld surface area percentage of only 4.2%.

### 2.7. Fluorescence Properties

**Figure 10:**
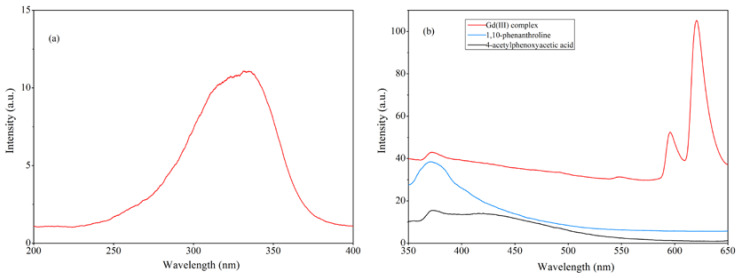
(**a**) The excitation spectrum of the Gd(III) complex and (**b**) the emission spectra of the Gd(III) complex, 4-acetylphenoxyacetic acid, and 1,10-phenanthroline in the ethanol solution. The excitation and emission slit widths are 5 nm.

The fluorescence properties of the Gd(III) complex, 4-acetylphenoxyacetic acid, and 1,10-phenanthroline in the ethanol solution were investigated. The excitation and emission spectra are shown in Figure 10. As shown in Figure 10a, the optimal excitation peak for the Gd(III) complex is 336 nm. The Gd(III) complex exhibits three emission peaks at 371 nm, 596 nm, and 620 nm, respectively, where the peak at 371 nm can be attributed to the emission peak of the ligand. The fluorescence emissions of the 4-acetylphenoxyacetic acid and 1,10-phenanthroline ligands in the ethanol solution were measured at the optimal excitation wavelength (336 nm) under the same conditions. Among them, the peak at 372 nm is the fluorescence emission of 4-acetylphenoxyacetic acid, and the peak at 370 nm is that of the 1,10-phenanthroline ligand. The fluorescence peak of the Gd(III) complex at 370 nm can be attributed to the emission peak of the 4-acetylphenoxyacetate and 1,10-phenanthroline ligands. The emission peaks at 596 nm and 620 nm of the Gd(III) complex belong to the ^5^D_0_ → ^7^F_1_ and ^5^D_0_ → ^7^F_2_ transitions, respectively, where the ^5^D_0_ → ^7^F_2_ transition at 620 nm is the strongest, with the next strongest being the ^5^D_0_ → ^7^F_1_ transition at 596 nm, indicating the best match between organic ligand triplet levels and the rare earth Gd(III) ion. Additionally, the coordination environment of the rare earth Gd(III) ion in the complex is favorable for efficient energy transfer.

### 2.8. Photocatalytic CO_2_ Reduction Activity of the Gd(III) Complex

**Figure 11:**
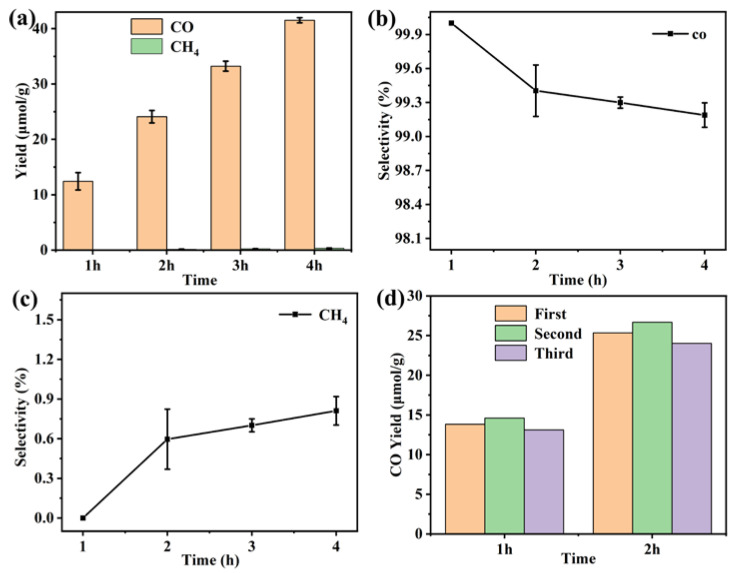
(**a**) Photocatalytic CO_2_ reduction performance, (**b**) CO and (**c**) CH_4_ selectivity of the Gd(III) complex, and (**d**) the cyclic experiments of photocatalytic CO_2_ reduction.

The photocatalytic CO_2_ reduction measurement of the Gd(III) complex was carried out to explore its application in the field of CO_2_ reduction. It can be observed in Figure 11 that the CO yield is 12.4 μmol/g in the first hour. After four hours of UV–vis light irradiation, the yield of CO reached 41.5 μmol/g. This indicates that the yield of CO gradually increases with the extension of reaction time, suggesting the presence of real catalytic activity of the Gd(III) complex. In addition, the CO selectivity is high compared to CH_4_; the value was more than 99%. The cyclic experiment photocatalytic CO_2_ reduction was carried out, and the results are shown in Figure 11d. It was found that the stability of the catalyst was reasonable. We studied photocatalytic CO_2_ reduction using the Yb (III) complex as the catalyst, and it showed good photocatalytic activity, with a CO yield and selectivity of 146 μmol/g and 97.9% after three hours of UV–vis light irradiation, indicating that the central metal ions have a significant effect on photocatalytic CO_2_ reduction [22]. Meanwhile, we also investigated photocatalytic CO_2_ reduction using the Gd(III) complex with different ligands, and they showed photocatalytic activity, with a CO yield and selectivity of 60.3 μmol/g and 100% after three hours of UV–vis light irradiation, indicating that the ligands also have an effect on photocatalytic CO_2_ reduction [23]. This provides a reference for us to continue photocatalytic CO_2_ reduction reactions using a complex as a catalyst in the future.

### 2.9. Catalytic Activity of the Gd(III) Complex

The prepared Gd(III) complex was explored for its catalytic performance as an oxidation catalyst using benzyl alcohol oxidation to benzaldehyde as a model reaction. The results are displayed in Table 3. The catalytic activity of the blank (without a catalyst) was very low, with a benzyl alcohol conversion of 6.5% for the selective benzyl alcohol oxidation at 120 °C within 2 h under 0.5 MPa of O_2_ using THF as a solvent. However, the conversion of benzyl alcohol increased significantly after adding the Gd(III) complex. The conversion of benzyl alcohol increased with increasing reaction temperature. The same phenomenon was observed when using 1,4-dioxane as a solvent (Table 3, entries 3–5). However, the benzaldehyde selectivity decreased with an increase in the reaction temperature. The solvents also displayed a remarkable effect on benzyl alcohol conversion, benzaldehyde selectivity, and the yields for the Gd(III) complex catalyst. The Gd(III) complex catalyst exhibited the highest catalytic activity (a yield of 45.7%) for selective benzyl alcohol oxidation to benzaldehyde in THF. Wang et al. [34] reported that the Zn(II) complex [ZnL_2_(H_2_O)_2_] (HL = 4-acetylbenzoic acid) showed good catalytic activity with a benzyl alcohol conversion rate of 85.6% and a benzaldehyde yield of 65.6% at 100 °C under 0.3 Mpa of O_2_ within 3 h. The benzyl alcohol conversion and benzaldehyde yield were 49.1% and 45.2% using the Ni(II) complex [Ni(L)_2_(H_2_O)_2_] (HL = 6-phenylpyridine-2-carboxylic acid) at 90 °C under 0.7 MPa within 2 h in THF [35]. The conversion of benzyl alcohol and the yield of benzaldehyde was 78.1% and 22.8% using the Zn(II) complex and ZnL_4_(Phen)_2_ catalyst (HL = 3-bromo-2-hydroxybenzaldehyde-pyridine-2-carbohydrazone, Phen = 1,10-phenanthroline) at 100 °C for 4 h under 5 bar of O_2_ [30]. The Gd(III) complex catalyst showed a lower conversion of benzyl alcohol than ZnL_4_(Phen)_2,_ but it showed a higher yield of benzaldehyde. Although the Gd(II) complex catalyst displayed lower catalytic activity than the [ZnL_2_(H_2_O)_2_] and [ZnL_2_(H_2_O)_2_] catalysts, it displayed good stability and could be reused at least three times with no drop in catalytic activity (Table 4).

The reusability of the Gd(III) complex catalyst in the benzyl alcohol oxidation reaction was tested at 120 °C within 2 h under 0.5 MPa of O_2_ using THF as the solvent. The results are shown in Table 4. Clearly, no drop in catalytic activity can be found using the Gd(III) complex catalyst in the first, second, and third runs. The Gd(III) complex catalyst displayed good stability for the oxidation of benzyl alcohol.

## 3. Experimental Section

### 3.1. Materials and Measurements

The reagents of gadolinium(III) nitrate hexahydrate, 4-acetylphenoxyacetic acid, NaOH, and 1,10-phenanthroline were used as received from the Jilin Chinese Academy of Sciences-Yanshen Technology Co., Ltd. (Jilin, China). The IR spectrum was obtained on a Tianjin Gangdong (Tianjin, China) FTIR-850 spectrophotometer (KBr discs, range 4000–400 cm^−1^), and the resolution and number of scans were 0.5 cm^−1^ and 8. The UV–vis spectra were acquired on a PERSEE T9 (Beijing, China) spectrophotometer equipped with quartz cuvettes with a 1 cm path length in the 190–700 nm region in a water solution. TG-DTA was performed on a HENVEN HCT-2 thermal analyzer (Beijing, China). The fluorescence measurements were acquired on a PE LS-55 fluorescence spectrophotometer in the 350–650 nm region, equipped with quartz cuvettes with a 1 cm path length (PerkinElmer, Waltham, MA, USA). The excitation and emission slit widths were 5 nm. The DFT calculations were performed to understand the electronic structure of the Gd(III) complex with the Gaussian 16 package [36]. The crystal structures were used to obtain the electron density distributions of the Gd(III) complex using the functional PBE0 [37] in combination with the pople basis set, 6-31G(d) [38], for C, H, N, O, and the large-core relativistic effective core potential (RECP) ECP53MWB [39] for Gd. The electron density distributions were visualized using the VMD package and Multiwfn program [40,41]. The Hirschfeld surface analysis of the Gd(III) complex was performed using CrystalExplorer software (https://crystalexplorer.net/, accessed on 10 April 2024) [42]. The crystal data of the Gd(III) complex were obtained on a Bruker CCD area detector (296.15 K, multi-scan, Cu at zero, SuperNova, Dual).

### 3.2. Synthesis of the Gd(III) Complex

4-Acetylphenoxyacetic acid (0.0971 g, 0.5 mmol), NaOH (0.020 g, 0.5 mmol), and gadolinium(III) nitrate hexahydrate (0.1239 g, 0.3 mmol) were added to the ethanol–water (*v*:*v* = 1:2) solution (30 mL) with stirring. 1,10-Phenanthroline (0.0405 g, 0.25 mmol) was added to the above mixture after 30 min. The mixture was then stirred for 3 h at 80 °C and stirred continuously for 2 h at room temperature. The product was collected via filtration. The colorless block crystals of the Gd(III) complex were obtained from the filtrate after 35 days. The elemental analysis calculated for [Gd_2_(L)_6_(Phen)_2_]·4H_2_O was C, 52.88%, H, 4.09%, N, 2.94%; Found: C, 52.61%, H, 4.37%, and N, 2.72%.

### 3.3. Crystal Structure Determination

The X-ray diffraction intensities of the Gd(III) complex were collected at 296.15 K from a block colorless crystal with dimensions of 0.15 mm × 0.12 mm × 0.10 mm. A total of 39,297 unique reflections were collected with a Bruker Smart CCD diffractometer in the range of 2.31° < *θ* < 23.65°using Olex2 [43] at 296.15 K. A total of 9953 reflections with *I* > 2(*σ*) were used in a structural solution, and the refinement was selected from 6998 independent reflections for X-ray diffraction and *R*_int_ = 0.0704. The structure was solved using the direct method with the SHELXS program [44] and refined with the SHELXL [45] program, respectively. The crystal data collection and handling of the Gd(III) complex are listed in Table 5.

The crystallographic data for the structure reported in this paper was deposited with the Cambridge Crystallographic Data Centre as supplementary publication No. CCDC 2347120. The CIF file can be obtained conveniently from the website: https://www.ccdc.cam.ac.uk/structures, accessed on 10 April 2024.

### 3.4. Photocatalytic CO_2_ Reduction Test

Firstly, 100 mL of deionized water, H_2_O, was added into a quartz reactor with vigorous stirring, and we controlled the temperature at 20 °C. Subsequently, the 50 mg Gd(III) complex catalyst was dispersed into the above solution. Then, the above mixed solution was bubbled using high-purity CO_2_ gas for 15 min. The reactor was sealed and began to perform the photocatalytic CO_2_ reduction experiment. The light source was the 300 W Xe arc lamp, which came from Beijing Trusttech Co., Ltd. (Beijing, China) The gas was let out every hour and tested via a gas chromatograph (Propark Q column, FID detector).

### 3.5. General Procedure for Benzyl Alcohol Oxidation

In a typical procedure, benzyl alcohol oxidation with O_2_ as the oxidant was carried out in a 20 mL stainless-steel high-pressure reactor equipped with a magnetic stirrer and thermoelectric couple. The mixture of the benzyl alcohol (1 mmol, 108.4 mg), solvents (tetrahydrofuran (THF) or dioxane, 7 mL), and the Gd(III) complex catalyst (25 mg) was added into the high-pressure reactor. Then, the high-pressure reactor was sealed and purged three times with O_2_. The reactor was subsequently pressurized with O_2_ to 0.5 MPa at room temperature. The high-pressure reactor was heated to the desired temperature (100–120 °C) and maintained for 2 h with vigorous stirring (2000 rpm). At the end of each run, the reactor was cooled down to room temperature and vented. The Gd(III) complex was filtered off via centrifugation (14,000 rpm), and the liquid products were analyzed via gas chromatography (GC-6890, Purkinje General instrument Co., Ltd., Beijing, China) with flame ionization detectors (FID) and a SE-54 capillary column (30 m × 0.25 mm × 0.25 mm). The conversion of benzyl alcohol and the selectivity of the benzaldehyde values represent the average of three experiments. The reuse of the Gd(III) complex catalyst for the catalytic oxidation of benzyl alcohol was performed within three runs to test the catalytic stability. The Gd(III) complex catalyst was separated from the mixture via centrifugation after each run and dried at 100 °C for 2 h in a drying oven. The catalyst mass was maintained at 25 mg for each run.

## 4. Conclusions

A new binuclear Gd(III) complex was synthesized and characterized using IR, UV–vis, TG-DSC, fluorescence, and single-crystal X-ray diffraction analyses. The Hirschfeld surface analysis and the density functional theory (DFT) calculations of the Gd(III) complex were performed. The catalytic activities of photocatalytic CO_2_ reduction and benzyl alcohol oxidation using the Gd(III) complex as a catalyst were measured. Based on the above results, a series of Gd(III) complexes could be designed and synthesized to optimize the catalytic activity of photocatalytic CO_2_ reduction and benzyl alcohol oxidation.

## Figures and Tables

**Table 1:** Important bond lengths (Å) and bond angles (°) for the Gd(III) complex.

Bond	*d*	Angle	(°)
Gd1-O1	2.424(3)	O1-Gd1-N1	89.00(12)
Gd1-O2	2.540(3)	O1-Gd1-N2	75.85(12)
Gd1-O7a	2.496(3)	O1-Gd1-O2	52.44(11)
Gd1-O8	2.365(3)	O1-Gd1-O7a	148.14(11)
Gd1-O8a	2.548(3)	O1-Gd1-O8a	148.81(11)
Gd1-O9	2.350(3)	O2-Gd1-N1	72.05(12)
Gd1-O10a	2.374(3)	O2-Gd1-N2	111.00(11)
Gd1-N1	2.545(4)	O2-Gd1-O8a	137.75(11)
Gd1-N2	2.599(4)	O7a-Gd1-N1	74.37(11)
		O7a-Gd1-N2	72.45(11)
		O7a-Gd1-O2	139.45(11)
		O7a-Gd1-O8a	51.53(10)
		O8-Gd1-N1	142.33(12)
		O8a-Gd1-N2	110.60(11)
		O8-Gd1-N2	148.26(12)
		O8-Gd1-O1	85.70(11)
		O8-Gd1-O2	75.30(11)
		O8-Gd1-O7a	123.92(10)
		O8-Gd1-O8a	73.44(11)
		O8-Gd1-O10a	74.18(11)
		O9-Gd1-N1	78.67(11)
		O9-Gd1-N2	136.76(11)
		O9-Gd1-O1	126.53(12)
		O9-Gd1-O2	74.38(11)
		O9-Gd1-O7a	77.35(11)
		O8-Gd1-O9	74.89(10)
		O9-Gd1-O8a	70.55(10)
		O9-Gd1-O10a	137.61(10)
		N1-Gd1-O10a	140.92(12)
		N2-Gd1-O10a	77.04(11)
		O1-Gd1-O10a	78.89(12)
		O2-Gd1-O10a	123.53(12)
		O7a-Gd1-O10a	96.91(12)
		O8a-Gd1-O10a	73.32(11)

Symmetry code: a:-*x*, 1-*y*, 1-*z.*

**Table 3:** The benzyl alcohol conversions, benzaldehyde selectivity, and yields for the Gd(III) complex in selective benzyl alcohol oxidation ^a^.

Entry	Solvent	Reaction Temperature (°C)	Conversion (%) ^b^	Selectivity (%) ^c^	Yield (%) ^d^
1	THF	100	3.3	90.3	3.0
2	THF	120	56.5	80.8	45.7
3	1,4-dioxane	100	5.5	81.3	4.5
4	1,4-dioxane	110	66.1	54.3	35.9
5	1,4-dioxane	120	87.4	15.0	13.1

^a^ The average standard deviations (three replicates) for the conversion, selectivity, and yield were 2.2%, 1.8%, and 2.1%, respectively; ^b^ The conversion was calculated using $C_{benzylalcohol}=\frac{n_{initial}-n_{afterreation}}{n_{initial}}\times 100\%$; ^c^ The selectivity was calculated using $S=\frac{n_{benzyldehyde}}{n_{benzylalcohol-initial}\times C_{benzylalcohol}}\times 100\%$; ^d^ The yield was calculated using $Y=C_{benzylalcohol}\times S\times 100\%$.

**Table 4:** The recyclability of the Gd(III) complex in benzyl alcohol oxidation ^a^.

Entry	Reaction Temperature (°C)	Conversion (%)	Selectivity (%)	Yield (%)
Fresh	120	56.5	80.8	45.7
Run 1	120	56.7	82.1	46.6
Run 2	100	55.6	82.5	45.9
Run 3	110	55.8	79.0	44.1

^a^ Reaction conditions: benzyl alcohol (1.0 mmol), THF (7.0 mL), Gd(III) complex (25.0 mg), 120 °C, 0.5 MPa, 2 h.

**Table 2:** The π-π stacking interaction data of the Gd(III) complex.

Ring-Ring	Symmetric Operation Code	Distance between Ring Centroids (Å)	Slippage (Å)
Cg2-Cg3	-*x*, 2-*y*, 1-*z*	3.726(3)	1.297
Cg2-Cg8		3.707(3)	1.210
Cg3-Cg2		3.726(3)	1.241
Cg8-Cg2		3.707(3)	1.220
Cg8-Cg8		3.725(2)	1.281

Cg2: N2-C6-C7-C10-C11-C12; Cg3: C4-C5-C6-C7-C8-C9; Cg8: N2-C6-C5-C4-C9-C8-C7-C10-C11-C12.

**Table 5:** The crystal data collection and handling of the Gd(III) complex.

Empirical Formula	C_84_H_78_Gd_2_N_4_O_28_
Formula weight	1906
Temperature/*K*	296.15
Crystal system	Triclinic
Space group	*P*-1
*a*/Å	12.305(3)
*b*/Å	12.816(3)
*c*/Å	14.551(3)
*α*/°	70.654(5)
*β*/°	80.229(5)
*γ*/°	65.394(5)
Volume/Å^3^	1967.1(8)
*Z*	1
ρ_calc_, mg/mm^3^	1.609
μ/mm^−1^	1.759
*S*	1.052
*F*(000)	962
Index ranges	−14 ≤ *h* ≤ 14, −15 ≤ *k* ≤ 15, −17 ≤ *l* ≤ 17
Reflections collected	39,298
Independent reflections	6998 [*R*(int) = 0.0704]
Data/restraints/parameters	6998/9/538
Goodness-of-fit on *F*^2^	1.047
Refinement method	Full-matrix least-squares on *F*^2^
Final *R* indexes [*I* ≥ 2σ (*I*)]	R_1_ = 0.0402, *wR*_2_ = 0.0698
Final *R* indexes [all data]	R_1_ = 0.0673, *wR*_2_ = 0.0790

## Data Availability

Data are contained within the article.

## Supplementary Materials

The following supporting information can be downloaded at: https://www.mdpi.com/article/10.3390/molecules29133039/s1, Figure S1: The calculated absorption spectrum of the ligands.

## References

[1] Liu X.J., Chen T.Q., Xue Y.H., Fan J.C., Shen S.L., Hossain M.S.A., Amin M.A., Pan L.K., Xu X.T., Yamauchi Y. (2022). Nanoarchitectonics of MXene/semiconductor heterojunctions toward artificial photosynthesis via photocatalytic CO_2_ reduction. Coord. Chem. Rev..

[2] Li M.D., Wang Z.M., Qi J., Yu R.B. (2023). Progress in the construction of metal oxide heterojunctions and their application in photocatalytic CO_2_ reduction. Chem. J. Chin. Univ..

[3] Gao X.Q., Cao L.L., Chang Y., Yuan Z.Y., Zhang S.X., Liu S.J., Zhang M.T., Fan H., Jiang Z.Y. (2023). Improving the CO_2_ Hydrogenation Activity of Photocatalysts via the Synergy between Surface Frustrated Lewis Pairs and the CuPt Alloy. ACS Sustain. Chem. Eng..

[4] Yin H.B., Li J.H. (2023). New insight into photocatalytic CO_2_ conversion with nearly 100% CO selectivity by CuO-Pd/H_x_MoO_3-y_ hybrids. Appl. Catal. B Environ..

[5] Heng Q.Q., Ma Y.B., Wang X., Wu Y.F., Li Y.Z., Chen W. (2023). Role of Ag, Pd cocatalysts on layered SrBi_2_Ta_2_O_9_ in enhancing the activity and selectivity of photocatalytic CO_2_ reaction. Appl. Surf. Sci..

[6] Shang X.F., Li G.J., Wang R.N., Xie T., Ding J., Zhong Q. (2023). Precision loading of Pd on Cu species for highly selective CO_2_ photoreduction to methanol. Chem. Eng. J..

[7] Yuan Z.M., Zhu X.L., Jiang Z.Y. (2023). Recent advances of constructing metal/semiconductor catalysts designing for photocatalytic CO_2_ hydrogenation. Molecules.

[8] Zhu X.L., Zong H.B., Pérez C.J.V., Miao H.H., Sun W., Yuan Z.M., Wang S.H., Zeng G.X., Xu H., Jiang Z.Y. (2023). Supercharged CO_2_ photothermal catalytic methanation: High conversion, rate, and selectivity. Angew. Chem. Int. Ed..

[9] Jiang M.P., Huang K.K., Liu J.H., Wang D., Wang Y., Wang X., Li Z.D., Wang X.Y., Geng Z.B., Hou X.Y. (2020). Magnetic-field-regulated TiO_2_ {100} facets: A strategy for C-C coupling in CO_2_ photocatalytic conversion. Chem.

[10] Qi M.Y., Lin Q., Tang Z.R., Xu Y.J. (2022). Photoredox coupling of benzyl alcohol oxidation with CO_2_ reduction over CdS/TiO_2_ heterostructure under visible light irradiation. Appl. Catal. B Environ..

[11] Yang M., Wang P., Li Y., Tang S., Lin X., Zhang H., Zhu Z., Chen F. (2022). Graphene aerogel-based NiAl-LDH/g-C_3_N_4_ with ultratight sheet-sheet heterojunction for excellent visible-light photocatalytic activity of CO_2_ reduction. Appl. Catal. B Environ..

[12] Zhao K., Zhao S., Gao C., Qi J., Yin H., Wei D., Mideksa M.F., Wang X., Gao Y., Tang Z. (2018). Metallic cobalt–carbon composite as recyclable and robust magnetic photocatalyst for efficient CO_2_ reduction. Small.

[13] Cai J., Lu J.Y., Chen Q.Y., Qu L.L., Lu Y.Q., Gao G.F. (2017). Eu-based MOF/graphene oxide composite: A novel photocatalyst for the oxidation of benzyl alcohol using water as oxygen source. New J. Chem..

[14] Tan T.H., Scott J., Ng Y.H., Taylor R.A., Aguey-zinsou K.F., Amal R. (2016). C–C Cleavage by Au/TiO_2_ during ethanol oxidation: Understanding bandgap photoexcitation and plasmonically mediated charge transfer via quantitative in situ drifts. ACS Catal..

[15] Zhou H., Xiao L.P., Liu X.N., Li S., Kobaya-shi H., Zheng X.M., Fan J. (2012). Defectless, layered organo-titanosilicate with superhydrophobicity and its catalytic activity in room-temperature olefin epoxidation. Chem. Commun..

[16] Xia W., Ren Y.Y., Liu J., Deng B.Y., Wang F. (2022). Non-synergistic photocatalysis of CO_2_-to-CO conversion by a binuclear complex of rigidly linking two cobalt catalytic centers. J. Photochem. Photobiol. A Chem..

[17] Jing H.W., Zhao L., Song G.Y., Li J.Y., Wang Z.Y., Han Y., Wang Z.X. (2023). Application of a mixed-ligand metal-organic framework in photocatalytic CO_2_ reduction, antibacterial activity and dye adsorption. Molecules.

[18] Xin X., Ma N., Hu C.Y., Liang Q., Bian Z.Y. (2019). Abundant manganese complex-anchored BiOI hybrid photocatalyst for visible light-driven CO_2_ reduction. Nano.

[19] Yasuomi Y., Takayuki O., Jun I., Shota F., Chinatsu T., Tomoya U., Taro T. (2019). Photocatalytic CO_2_ reduction using various heteroleptic diimine-diphosphine Cu(I) complexes as photosensitizers. Front. Chem..

[20] Fu Z.C., Mi C., Sun Y., Yang Z., Xu Q.Q., Fu W.F. (2019). An unexpected iron (II)-based homogeneous catalytic system for highly efficient CO_2_-to-CO conversion under visible-light irradiation. Molecules.

[21] Sakakibara N., Shizuno M., Kanazawa T., Kato K., Yamakata A., Nozawa S., Ito T., Terashima K., Maeda K., Tamaki Y. (2023). Surface-specific modification of graphitic carbon nitride by plasma for enhanced durability and selectivity of photocatalytic CO_2_ reduction with a supramolecular photocatalyst. ACS Appl. Mater. Interfaces.

[22] Tai X.S., Wang Y.F., Wang L.H., Yan X.H. (2023). Synthesis, structural characterization, hirschfeld surface analysis and photocatalytic CO_2_ reduction of Yb(III) complex with 4-acetylphenoxyacetic acid and 1,10-phenanthroline ligands. Bull. Chem. React. Eng. Catal..

[23] Wang L.H., Tai X.S. (2023). Synthesis, structural characterization, hirschfeld surface analysis and photocatalytic CO_2_ reduction activity of a new dinuclear Gd(III) complex with 6-phenylpyridine-2-carboxylic acid and 1,10-phenanthroline ligands. Molecules.

[24] Meena B.I., Lakk-Bogáth D., Kaizer J. (2021). Effect of redox potential on manganese-mediated benzylalcohol and sulfide oxidation. Comptes Rendus Chim..

[25] Lu M.Y., Hu X.Y., Hu Q.X., Yang H.C., Lai D.L., Yan X.L., Feng R., Zhao G.F. (2021). Selective oxidation of benzyl alcohol to benzaldehyde with air using ZIF-67 derived catalysts. Colloids Surf. A Physicochem. Eng. Asp..

[26] Shoair A.F., El-Bindary A.A., Abd El-Kader M.K. (2017). Structural and catalytic properties of some azo-rhodanine ruthenium(III) complexes. J. Mol. Struct..

[27] Yang X.J., Mao J.C., Zhang H., Zhang Y., Mao J.H. (2018). Copper-catalyzed aerobic oxidation reaction of benzyl alcohol in water under base-free condition. Chin. J. Org. Chem..

[28] Urgoitia G., Galdón G., Churruca F., Sanmartin R., Herrero M.T., Domínguez E. (2018). Aerobic oxidation of secondary benzyl alcohols catalyzed by phosphinite-based palladium pincer complexes. Environ. Chem. Lett..

[29] Tai X.S., Yan X.H., Wang L.H. (2024). Synthesis, structural characterization, hirschfeld surface analysis, density functional theory, and photocatalytic CO_2_ reduction activity of a new Ca(II) complex with a bis-Schiff base ligand. Molecules.

[30] Wang L.H., Wang Z.J., Zhao M.L., Tai X.S., Ouyang J., Li Y.F., Zhang W., Jia W.L. (2021). Synthesis, crystal structure of tetra-nuclear macrocyclic Zn (II) complex and its application as catalyst for oxidation of benzyl alcohol. Bull. Chem. React. Eng. Catal..

[31] Wang L.H., Tai X.S., Liu L.L., Li P.F. (2017). Synthesis, crystal structure and catalytic activity of a novel Ba(II) complex with pyridine-2-carboxaldehyde-2-phenylacetic acid hydrazone ligand. Crystals.

[32] Wu Y.X., Zhou J.H., Zhao H.B., Hu Y.C. (2005). Characterization and analysis of phenanthroline derivatives. J. Cent. South For. Univ..

[33] Becke A.D. (1993). A new mixing of Hartree-Fock and local density-functional theories. J. Chem. Phys..

[34] Wang L.H., Tai H.W. (2023). Synthesis, structural characterization, DFT, hirschfeld surface and catalytic activity of a new Zn(II) complex of 4-acetylbenzoic acid. Bull. Chem. React. Eng. Catal..

[35] Wang L.H., Kong F.Y., Tai X.S. (2022). Synthesis, structural characterization of a new Ni(II) complex and its catalytic activity for oxidation of benzyl alcohol. Bull. Chem. React. Eng. Catal..

[36] Frisch M.J., Trucks G.W., Schlegel H.B., Scuseria G.E., Robb M.A., Cheeseman J.R., Calmani G., Barone V., Petersson G.A., Nakatsuji H. (2019). Gaussian 16, Revision C.02.

[37] Adamo C., Barone V. (1999). Toward reliable density functional methods without adjustable parameters: The PBE0 model. J. Chem. Phys..

[38] Francl M.M., Pietro W.J., Hehre W.J., Binkley J.S., Gordon M.S., DeFrees D.J., Pople J.A. (1982). Self-consistent molecular orbital methods. XXIII. A polarization-type basis set for second-row elements. J. Chem. Phys..

[39] Dolg M., Stoll H., Savin A., Preuss H. (1989). Energy-adjusted pseudopotentials for the rare Earth elements. Theor. Chim. Acta.

[40] Humphrey W., Dalke A., Schulten K. (1996). VMD: Visual molecular dynamics. J. Mol. Graph..

[41] Lu T., Chen F. (2012). Multiwfn: A multifunctional wavefunction analyzer. J. Comput. Chem..

[42] Spackman P.R., Turner M.J., McKinnon J.J., Wolff S.K., Grimwood D.J., Jayatilaka D., Spackman M.A. (2021). CrystalExplorer:a program for Hirshfeld surface analysis, vis-ualization and quantitative analysis of molecular crystals. J. Appl. Crystallogr..

[43] Dolomanov O.V., Bourhis L.J., Gildea R.J., Howard J.A.K., Puschmann H. (2009). OLEX2: A complete structure solution, refinement and analysis program. J. Appl. Crystallogr..

[44] Sheldrick G.M. (2008). A short history of SHELX. Acta Crystallogr..

[45] Sheldrick G.M. (2015). Crystal structure refinement with SHELXL. Acta Crystallogr..

