# Supplementary material for: The Synthesis, Structural Characterization, and DFT Calculation of a New Binuclear Gd(III) Complex with 4-Aacetylphenoxyacetic Acid and 1,10-Phenanthroline Ligands and Its Roles in Catalytic Activity
Source: Molecules. 2024 Jun 26;29(13):3039. doi: 10.3390/molecules29133039 (PMC11243657; doi:10.3390/molecules29133039)
Supplement: Supplementary file 1 [file molecules-29-03039-s001.zip › molecules-3021239-supplementary.pdf]

The Synthesis, Structural Characterization, and DFT Calculation of a New Binuclear Gd(III) Complex with 4-Aacetylphenoxyacetic Acid and 1,10-Phenanthroline Ligands and Its Roles in Catalytic Activity

Ying Liu [1], Xiao Tang [2], Xi-Hai Yan [1], Li-Hua Wang [3], Xi-Shi Tai [1,*], Mohammad Azam [4,*] and Dong-Qiu Zhao [5,*]

[1] College of Chemistry and Chemical Engineering, Weifang University, Weifang 261061, China; ydliuying@163.com (Y.L.); yan7899@126.com (X.-H.Y.)
[2] College of Science, Institute of Materials Physics and Chemistry, Nanjing Forestry University, Nanjing 210037, China; xiaotang@njfu.edu.cn
[3] College of Biology and Oceanography, Weifang University, Weifang 261061, China; wanglihua929@163.com
[4] Department of Chemistry, College of Science, King Saud University, Riyadh 11451, Saudi Arabia
[5] School of Physics and Electric Engineering, Anyang Normal University, Anyang 455000, China
* Correspondence: taixs@wfu.edu.cn (X.-S.T.); azam_res@yahoo.com (M.A.); dqzhao@aynu.edu.cn (D.-Q.Z.); Tel.: +86-536-8785286 (X.-S.T.); Fax: +86-536-8785286 (X.-S.T.)

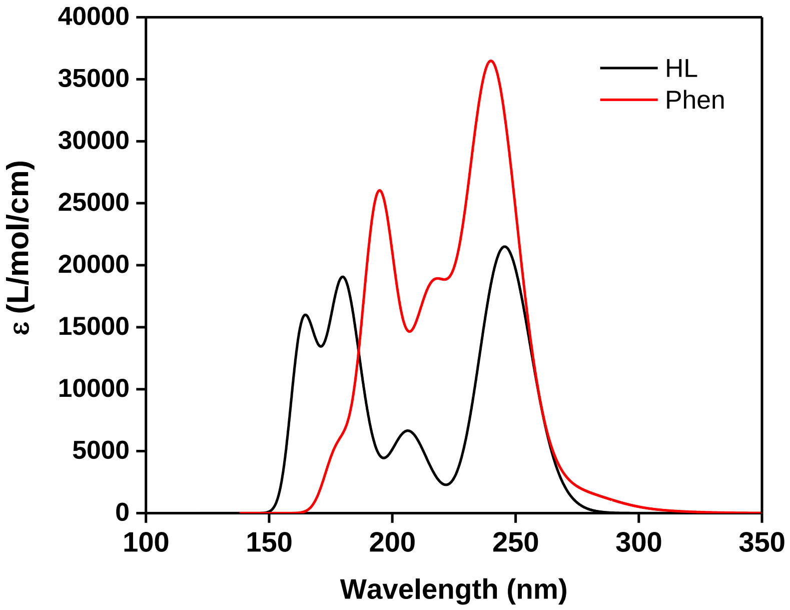

Figure s1. The calculated absorption spectrum of the ligands
